# Five new species of *Dolichomitus* Smith from the tropical Andes, with a key for the South American species (Hymenoptera, Ichneumonidae, Pimplinae)

**DOI:** 10.3897/zookeys.937.51361

**Published:** 2020-06-01

**Authors:** Rodrigo O. Araujo, Diego G. Pádua, Jorge Jaramillo, Luis A. Mazariegos

**Affiliations:** 1 Centro de Investigación de Estudios Avanzados del Maule, Vicerrectoría de Investigación y Postgrado, Universidad Católica del Maule, Avenida San Miguel, 3605, Talca, Chile; 2 Laboratorio de Ecología de Abejas, Departamento de Ciencias Biológicas y Químicas, Facultad de Ciencias Básicas, Universidad Católica del Maule, Avenida San Miguel, 3605, Talca, Chile; 3 Programa de Pós-Graduação em Entomologia, Instituto Nacional de Pesquisas da Amazônia, Manaus, Amazonas, Brazil; 4 The Hummingbird Conservancy, Mesenia-Paramillo nature reserve, Jardin, Antioquia, Colombia

**Keywords:** Colombia, Darwin wasps, Ephialtini, Mesenia-Paramillo, Neotropical, ovipositor, parasitoid wasps, taxonomy

## Abstract

*Dolichomitus* Smith is a widely distributed pimpline genus with more than seventy known species. There are eight species previously reported from South America: *D. annulicornis* (Cameron), *D. bivittatus* Townes, *D. hypermeces* Townes, *D. jatai* Loffredo & Penteado-Dias, *D. longicauda* Smith, *D. megalourus* (Morley), *D. moacyri* Loffredo & Penteado-Dias and *D. zonatus* (Cresson). In this paper, we describe five new species: *D. mariajosae* Araujo & Pádua, **sp. nov.**, *D. menai* Araujo & Pádua, **sp. nov.**, *D. orejuelai* Araujo & Pádua, **sp. nov.**, *D. pimmi* Araujo & Pádua, **sp. nov.**, and *D. rendoni* Araujo & Pádua, **sp. nov.** All have been collected in cloud forests in the Colombian tropical Andes. An illustrated key to the South American species of the genus is also provided.

## Introduction

The Darwin wasps are among the most species-rich branches of the tree of life, with approximately 25,000 species belonging to 41 subfamilies (Yu et al. 2016; Bennett et al. 2019; Klopfstein et al. 2019). At the same time, it is one of the groups for which our taxonomic knowledge most severely lags behind their actual diversity (Klopfstein et al. 2019). The Pimplinae are one of the best studied subfamilies in the Neotropical Region, mainly due to the taxonomic revisions performed by Ian Gauld (e.g. Gauld 1991; Gauld et al. 1998, 2002). However, recent studies continue to reveal new species of this subfamily in this region (e.g. Gómez et al. 2014; Sääksjärvi et al. 2015; Bordera et al. 2016; Palacio et al. 2018; Bordera and Palacio 2019; Palacio et al. 2019; Bordera et al. 2019; Pádua et al. 2020), as well as expanding the distribution records for some of it genera (Pádua et al. 2019a, 2019b). These studies indicate that unsampled areas have great potential for revealing new species to science, especially considering that few areas of South America were sampled adequately (Gómez et al. 2009).

Because of their long ovipositors and large bodies, *Dolichomitus* wasps are one of the most conspicuous and well-known genera in the Ichneumonidae, with more than seventy known species in the world (Matsumoto 2018). There are 15 species in the Neotropical region, with eight species occurring in South America: *D. annulicornis* (Cameron, 1886); *D. bivittatus* Townes, 1975; *D. hypermeces* Townes, 1975; *D. jatai* Loffredo & Penteado-Dias, 2012; *D. longicauda* Smith, 1877; *D. megalourus* (Morley, 1914); *D. moacyri* Loffredo & Penteado-Dias, 2012 and *D. zonatus* (Cresson, 1874), besides two subspecies (Yu et al. 2016). From a biological point of view, they are pupal ectoparasitoids known to attack mainly coleopteran larvae that bore in dead wood, especially Cerambycidae and, less commonly, Curculionidae, Melandryidae, and Scolytidae (Townes and Townes 1960; Fitton et al. 1988).

Herein we describe five new species of *Dolichomitus* and provide the first identification key for the species present in South America.

## Materials and methods

Morphological terminology follows Broad et al. (2018). The information contained in “Type Material” sections corresponds to the specimen labels *verbatim*. The specimens will be deposited in the Universidad de los Andes (**UNIANDES**) entomological collection in Bogotá, Colombia, under “ANLA collection permit of specimens of the biological diversity” (curator Emilio Rialpe).

The specimens examined in this study were all collected with sweeping net and manual collection during an inventory carried out in the Mesenia-Paramillo Natural Reserve (5°29'46.1"N, 75°53'20.5"W) between February and December 2019, in the department of Antioquia, Colombia. This is a private conservation area of 3,500 ha located in the western Andes, more specifically in the Northwestern Andean montane forest ecoregion, which is among the most diverse regions on the planet (see Pádua et al. 2019a).

Drawings were adapted from Gauld (1991) and vectorized by Adobe Illustrator. Photographs were prepared using a Canon EOS 5D Mark IV and 5DSR, and Canon EF 100mm f/2.8 IS USM and MP-E 65mm f/2.8 1-5X macro lenses and edited with Adobe Photoshop (v. CS5), or a Leica DMC4500 digital camera attached to a Leica M205A stereomicroscope and combined using the software Leica Application Suite V4.10.0. All measurements were rounded to the nearest 0.05.

## Taxonomy

### Dolichomitus

Taxon classificationAnimaliaHymenopteraIchneumonidae

Smith, 1877

E71A4DE4-4C4D-541D-9567-679B9FD0D12C

Closterocerus Hartig, 1847: 18. Type-species: Closterocerus sericeus Hartig, by monotypy. [Homonym of Closterocerus Westwood, 1833].
Dolichomitus Smith, 1877: 411. Type-species: Dolichomitus longicauda Smith, by monotypy.
Mesoephialtes Schmiedeknecht, 1906: 1014. Type-species: Mesoephialtes coracinus Schmiedeknecht (= Pimpla zonata Cresson), by monotypy.
Diclosterocerus Viereck, 1914: 45. [Replacement name for Closterocerus Hartig].

#### Diagnosis.

The genus can be identified by the following combination of character states: (1) clypeus not divided in anterior and posterior parts; (2) clypeal margin narrow, apically bilobate; (3) occipital carina more or less complete, mediodorsally dipped, sometimes weak; (4) propodeum with a trace of the lateromedian longitudinal carinae discernible anteriorly; (5) fore wing with 3*rs-m* present; (6) hind wing with distal abscissa of *CU* present, joining *cu-a* either closer to 1*AA* than to *M*, or closer to *M* than to *AA*; (7) male (in most species) with middle coxa modified in one or two concavities on outer surface and basal, apical and/or centrally tubercles; (8) female with basal lobe on tarsal claws; (9) tergite II with oblique groves cutting off depressed triangular areas anterolaterally; (10) male with sternite IX transverse, posteriorly slightly convex; (11) ovipositor 3.00–13.00× as long as hind tibia; (12) upper valve smooth and lower valve of ovipositor laterally expanded to partially enclose upper valve.

### Key to the South American species of *Dolichomitus*

[The males of *D. bivittatus* Townes, *D. hypermeces* Townes, *D. longicauda* Smith, *D. mariajosae* sp. nov., *D. menai* sp. nov., *D. orejuelai* sp. nov., *D. pimmi* sp. nov. and *D. rendoni* sp. nov. are unknown].

**Table:** 

1	Female	**2**
–	Male	**14**
2	Ovipositor sheath long, < 1.50× as long as body	**3**
–	Ovipositor sheath very long, > 3.00× as long as body	**11**
3	Mesosoma reddish brown or reddish black with white, yellow or black marks (Figs 1C, D, 4A, B, 6A, B)	**4**
–	Mesosoma black or yellow or orange yellow or yellowish brown with black marks (Figs 1A, B, 2A, B, 3A, B, 5A, B)	**6**
4	Head almost yellow with vertex brown; occipital region, central frons and mandible black; fore wing yellowish with anterior margin more fuscous, pterostigma yellow (Fig. 1L)	***D. moacyri* Loffredo & Penteado-Dias**
–	Head almost reddish black, without yellow marks; fore wing entirely yellowish, pterostigma light or dark brown (Figs 4G, 6G)	**5**
5	Tegula reddish black (Fig. 4D, E); areolet not petiolate; pterostigma light brown (Fig. 4G); hind wing with vein *cu-a* ca. 2.25× as long as proximal abscissa of *CU*; fore and mid legs with color pattern mostly red and reddish black; metasoma mostly yellowish brown (Fig. 4A, B)	***D. orejuelai* sp. nov.**
–	Tegula white (Fig. 6D, E); areolet slightly petiolate; pterostigma dark brown (Fig. 6G); hind wing with vein *cu-a* ca. 1.20× as long as proximal abscissa of *CU*; fore and mid legs with color pattern mostly white; metasoma mostly dark brown (Fig. 6A, B)	***D. rendoni* sp. nov.**
6	Fore wing iridescent or hyaline or yellowish, but always with apex black (Figs 2G, 3G, 5G)	**7**
–	Fore wing entirely yellowish or yellowish with anterior margin more strongly yellowish (Fig. 1I, J, L)	**9**
7	Malar space 0.55× as long as basal mandibular width; head mostly black (Fig. 3C); full-spectrum iridescent wings with strongly contrasting apical darkened area that at least covers completely the fourth submarginal cell and third discal cell, pterostigma black (Fig. 3G); metasoma mostly black shinning (Fig. 3A, B, F)	***D. menai* sp. nov.**
–	Malar space 0.30× as long as basal mandibular width; head mostly yellowish (Figs 2C, 5C); wings hyaline or yellowish with strongly contrasting apical darkened area that covers only the distal half of fourth submarginal cell, pterostigma dark or light brown (Figs 2G, 5G); metasoma mostly yellowish with lateral spots and an anterior dorsal longitudinal stripe on tergite I (Figs 2F, 5F)	**8**
8	Fore wing hyaline with pterostigma dark brown (Fig. 2G); hind wing with proximal abscissa of *CU* inclivous; tergites II–IV yellow with a dorsolateral mark on anterior margin and a band in the posterior margin black; ovipositor ca. 4.40× as long as hind tibia; ovipositor sheath ca. 4.20× as long as hind tibia (Fig. 2A)	***D. mariajosae* sp. nov.**
–	Fore wing yellowish with pterostigma light brown (Fig. 5G); hind wing with proximal abscissa of *CU* vertical; tergites II–IV yellow with posterior margins black; ovipositor ca. 3.40× as long as hind tibia; ovipositor sheath ca. 3.00× as long as hind tibia (Fig. 5A)	***D. pimmi* sp. nov.**
9	Metasoma yellowish brown with tergites III+ or IV+ blackish (Fig. 1F)	***D. jatai* Loffredo & Penteado-Dias**
–	Metasoma mostly light brown; tergite I–III subapically yellow with lateral posterior of margin brown to black; rest of tergites with posterior margins brown. (Fig. 1E)	**10**
10	Propodeum with a central and anterior smooth area, strongly and evenly broadened posteriorly so that near to the hind margin is more than twice as broad as anteriorly (Fig. 1N), generally with this area partly to completely black and the slightly raised part lateral to it yellow; hind wing with distal abscissa of *CU* joining *cu-a* almost equidistant between *AA* and *M*, or sometimes closer to *AA*	***D. annulicornis* (Cameron)**
–	Propodeum with a central and anterior smooth area, only slightly expanded posteriorly so that near the hind margin is less than twice as broad as anteriorly (Fig. 1O), generally with this area black, but with parallel black stripes on the slightly raised, yellow, lateral part; hind wing with distal abscissa of *CU* joining *cu-a* obviously closer to *M* than to *AA*	***D. zonatus* (Cresson)**
11	Fore wing black with pterostigma yellow (Fig. 1K)	***D. megalourus* (Morley)**
–	Fore wing yellow with two black bands or entirely infumate or brown with a broad pale yellowish-brown band on apex	**12**
12	Fore wing with two black bands (Fig. 1M)	***D. bivittatus* Townes**
–	Fore wing entirely infumate or brown with a broad pale yellowish brown band on apex	**13**
13	Body black; ovipositor sheath 7.00–8.10× as long as body; fore wing brown with a broad pale yellowish brown band on apex	***D. hypermeces* Townes**
–	Body black with metasomal tergites I–II yellow; ovipositor sheath 4.50–7.00× as long as body; fore wing infumate	***D. longicauda* Smith**
14	Middle coxa modified in two concavities on outer surface or a distinct basal prominence (Fig. 1P, Q)	**15**
–	Middle coxa evenly convex (Fig. 1R)	**16**
15	Middle coxa modified in two concavities on outer surface (Fig. 1P)	***D. annulicornis* (Cameron)**
–	Middle coxa with a distinct basal prominence on outer surface (Fig. 1Q)	***D. zonatus* (Cresson)**
16	Fore wing black with pterostigma yellow (Fig. 1K)	***D. megalourus* (Morley)**
–	Fore wing yellowish or yellowish with anterior margin slightly fuscous (Fig. 1J, L)	**17**
17	Mesosoma yellow with mesoscutum with three black stripes, the anterior margin and posterior lateral margin of the propodeum with a narrow black stripe, pronotum, mesopleuron and metapleuron with posterior margin black (Fig. 1B); metasoma yellow with tergites III+ or IV+ black (Fig. 1F)	***D. jatai* Loffredo & Penteado-Dias**
–	Mesosoma reddish brown, with three black stripes on mesoscutum, dorselum yellow, and propleuron black (Fig. 1D); metasoma with tergite I brownish and tergites II+ reddish brown (Fig. 1H)	***D. moacyri* Loffredo & Penteado-Dias**

**Figure 1.**
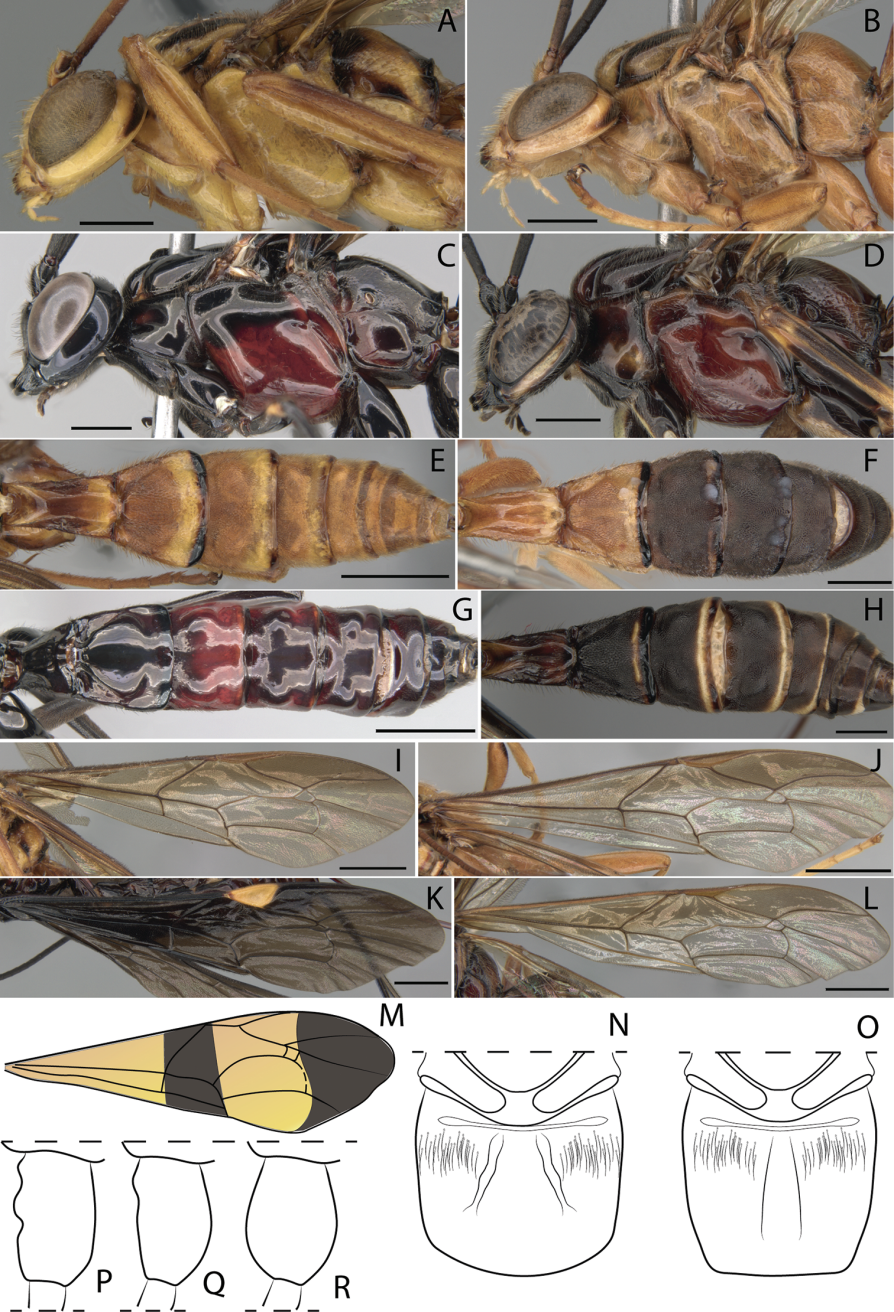
**A–R***Dolichomitus* species **A–D** head and mesosoma, lateral view: **A***D. annulicornis***B***D. jatai***C***D. megalourus***D***D. moacyri***E–H** metasoma, dorsal view: **E***D. annulicornis***F***D. jatai***G***D. megalourus***H***D. moacyri***I–M** fore wing **I***D. annulicornis***J***D. jatai***K***D. megalourus***L***D. moacyri***M***D. bivittatus***N, O** propodeum, dorsal view: **N***D. annulicornis***O***D. zonatus***P–R** mid coxa, lateral view, ♂ (Modified of Gauld 1991): **P***D. annulicornis***Q***D. zonatus***R***D. megalourus*. Scale bars: 1.00 mm (**A, B, C, D, F, H**); 2.00 mm (**E, G, I, J, K, L**).

### Dolichomitus mariajosae

Taxon classificationAnimaliaHymenopteraIchneumonidae

Araujo & Pádua sp. nov.

A8821470-62B7-5C67-B23F-E3C60D9DFDB9

http://zoobank.org/9EFFAC5D-B6B0-447D-94FF-F6835D03EDAA

Fig. 2A–G

#### Diagnosis.

*Dolichomitus mariajosae* sp. nov. may be distinguished from other Neotropical species by the combination of the following characteristics: general color pattern (yellow with various specifics black marks); malar space 0.30× as long as basal mandibular width; areolet not petiolate; wings hyaline with strongly contrasting apical darkened area, pterostigma dark brown; hind wing with proximal abscissa of *CU* inclivous; ovipositor sheath ca. 1.30× as long as body, and ca. 4.20× as long as hind tibia.

#### Description.

**Figure 2.**
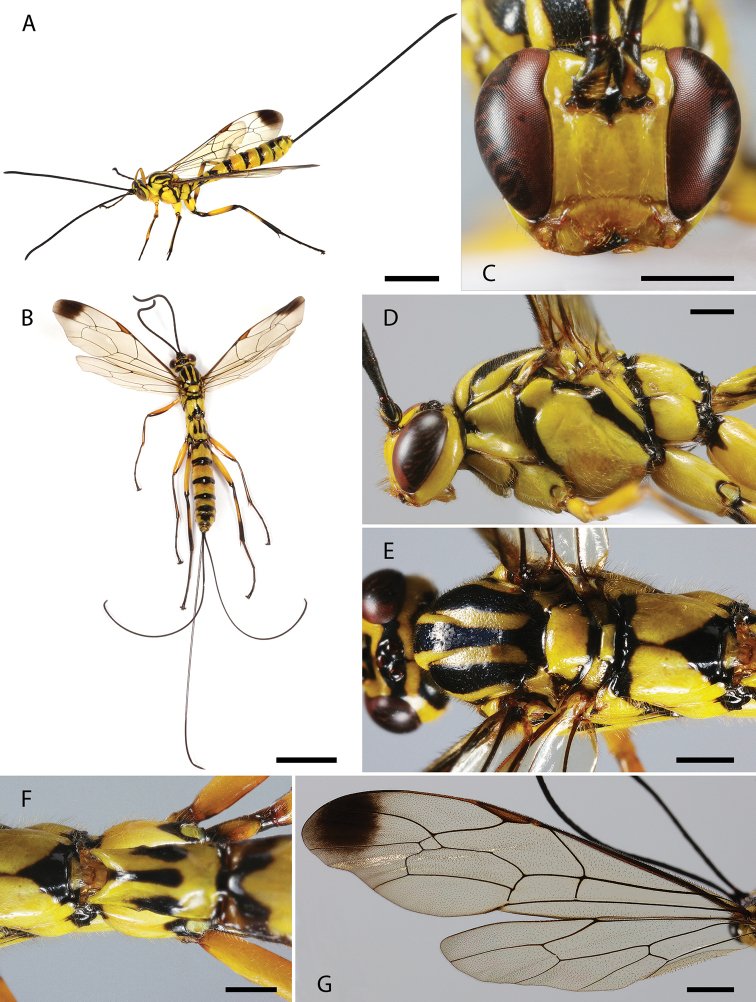
**A–G***Dolichomitus mariajosae* sp. nov. (holotype female): **A** habitus in lateral view (*in vivo*) **B** habitus in dorsal view **C** head in frontal view **D** head and mesosoma in lateral view **E** mesosoma in dorsal view **F** first tergite in dorsal view **G** wings. Scale bars: 5.00 mm (**A, B**); 1.00 mm (**C, D, E, F**); 2.00 mm (**G**).

**Holotype female** (Fig. 2A–G). Approximate body length (without ovipositor): 15.90 mm; fore wing length: 14.00 mm.

***Head.*** Antenna with 34 flagellomeres, first flagellomere 3.80× as long as width. Gena smooth with setiferous punctures, 0.50× as long as eye (Fig. 2D), in frontal view almost straight and moderately constricted below eyes (Fig. 2C). Vertex smooth and shiny, with isolated setiferous punctures. Posterior ocellus separated from eye 1.35× its maximum diameter. Distance between hind ocelli 1.25× maximum diameter of posterior ocellus. Face with fine, setiferous punctures. Clypeal sulcus slightly curved. Clypeus 3.25× as broad as medially long, almost flat. Clypeus with long erect setae on its surface and small setae across all its margins. Anterior tentorial pits conspicuous. Malar space 0.30× as long as basal mandibular width. Mandible bidentate, 2.05× as long as basal width (front view).

***Mesosoma.*** Pronotum polished, with fine and scattered setiferous punctures. Epomia present. Mesoscutum shiny, with moderately dense setiferous punctures. Notauli deep, reaching ca. 0.30–0.40 of length of mesoscutum. Mesopleuron shiny, with relatively dense setiferous punctures. Epicnemial carina strong. Metapleuron shiny, with scattered setiferous punctures, ca. 1.45× as long as height. Submetapleural carina strong, enlarged anteriorly, reaching ca. 0.40 metapleuron length, its anterior end slightly curved up. Propodeum shiny, with fine and scattered setiferous punctures, denser laterally, in dorsal view 1.15× as long as medially wide. Propodeal spiracle elliptic. Pleural carina complete and strong, culminating posteriorly in a small propodeal crest (Fig. 2D). Hind leg with femur ca. 6.50× as long as height and ca. 0.70× as long as tibia. Fore wing with vein *1cu-a* more or less interstitial to *M*&*Rs*; areolet 1.50× as wide as height; vein *1cu-a* and vein *2m-cu* slightly curved. Hind wing with vein *cu-a* ca. 2.10× as long as proximal abscissa of *CU*; vein *cu-a* reclivous and straight; proximal abscissa of *CU* inclivous; distal abscissa of *CU* present, reaching wing margin (Fig. 2G).

***Metasoma.*** Tergite I ca. 1.75× as long as posteriorly wide, shiny, with fine and relatively dense setiferous punctures, more extended laterally (Fig. 2F); spiracle near its anterior 0.40; dorsolateral carinae of first metasomal tergite weak, present on petiole and postpetiole. Posterior membranous section of first metasomal sternite ca. 0.50 of length of tergite. Tergite II ca. 1.20× as long as posteriorly wide, shiny, with fine and relatively dense setiferous punctures, more extended laterally and posteriorly. Ovipositor slender, evenly down curved at distal 0.15, ca. 4.40× as long as hind tibia (Fig. 2A); upper valve of ovipositor smooth; apex of ovipositor with expanded area of lower valve bearing ca. 11 teeth, with most proximal 5 vertical; ovipositor sheath ca. 1.30× as long as body, and ca. 4.20× as long as hind tibia, bearing fine dense hairs which are ca. 0.75× as long as width of sheath.

***Color.*** Head mostly yellow with mandibles apically, frons, stemmaticum (extending to outer orbit dorsally), a narrow longitudinal stripe on vertex, dorsal half of occipital carinae, scape, pedicel, and flagellum black; scape ventrally and malar space infuscate. Mesosoma mostly yellow with anterior, posterior, and inner margin of propleuron, a narrow longitudinal stripe on pronotal collar dorsally (dorsally extending to pronotum), anterior margin of pronotum (connecting with mesoscutum), posterior margin of pronotum, subtegular ridge, lateral and central longitudinal stripes on mesoscutum, two marks on the lateral margins of mesoscutum (just above tegula), scuto-scutellar groove, posterior margin of scutellum, anterior margin and axilla posteriorly, anterior and posterior margins of metanotum, epicnemium (with a small rounded projection toward speculum), anterior and dorsal margin of mesopleuron, mesopleural furrow, anterior margin of mesepisternum, anterior and posterior margin of metapleuron, submetapleural carina anteriorly, anterior and posterior margin of propodeum, a triangle mark dorsally based on the posterior margin that projects itself towards the anterior margin through a narrow longitudinal stripe, black. Fore leg mostly yellow with a dorsal spot on coxa, posterior margin on coxa, anterior spot on trochanter, femur ventrally, tibia dorsally, tarsus black. Mid leg mostly yellow with anterior and posterior margin of coxa, anterior margin of trochanter, ventral stripe on femur, dorsal stripe on tibia, tarsus, black; trochantellus infuscate. Hind leg mostly yellow with anterior (projecting ventrally), posterior (projecting dorsally) margins of coxa, anterior and posterior margins trochanter, trochanter ventrally, trochantellus, anterior and posterior margins of femur, ventral stripe on femur, anterior and posterior margins of tibia, dorsal stripe on tibia, tarsus black (Fig. 2A, B, F). Wings hyaline with strongly contrasting apical darkened area that covers only the distal half of fourth submarginal cell, pterostigma dark brown (Fig. 2G). Metasoma mostly yellow, tergite I with lateral and posterior margins, lateral spots (near spiracle) and a median longitudinal stripe reaching ca. 0.70 of tergite I; a dorsolateral mark on anterior margin and a band on the posterior margin of tergites II–IV, a dorsal mark on tergites VI–VIII, ventral corner of the posterior margin of tergites V–VI, black. Ovipositor dark brown and ovipositor sheath black.

**Male.** Unknown.

#### Type material.

***Holotype.*** 1 ♀, Colombia, Jardín, Antioquia, La Lucrecia, Mesenia-Paramillo nature reserve (2400m elevation), 5°30'50.61"N, 75°50'32.02"W, entomological net, 06–I–2020, coll. Jaramillo, J. (UNIANDES).

#### Distribution.

Colombia.

#### Etymology.

The specific epithet is in honor of Maria Jose Valencia, daughter of Carlos Eduardo Valencia, Colombian entrepreneur, who supports conservation initiatives in the Andes and Chocó ecoregions, and enjoys the natural world and the challenges of exploring the outdoors.

#### Biological note.

Host unknown.

#### Comments.

*Dolichomitus mariajosae* sp. nov. is most similar to the *D. zonatus* (Cresson, 1874), *D. cantillanoi* Gauld, 1991, and *D. annulicornis* (Cameron, 1886) mainly for the color pattern of the body yellowish with black marks, but this new species differs mainly for the fore wing with black spot in the apex (yellowish with anterior margin strongly yellow in *D. annulicornis* and *D. zonatus*, and entirely yellowish in *D. cantillanoi*).

### Dolichomitus menai

Taxon classificationAnimaliaHymenopteraIchneumonidae

Araujo & Pádua sp. nov.

B0E965FE-96F6-50F8-A5BE-6153BABFA71C

http://zoobank.org/AF3875BF-C9F5-411E-BA97-52D608350FEB

Fig. 3A–G

#### Diagnosis.

*Dolichomitus menai* sp. nov. may be distinguished from other Neotropical species by the combination of the following characteristics: head mostly black with clypeus predominantly dark brown, anterior margin of clypeus, inner orbit, frontal orbit, outer orbit yellow; fore leg mostly black with ventral surfaces of femur and tibiae yellow; wings iridescent rainbow colors with strongly contrasting subapical darkened area, pterostigma black; areolet not petiolated; malar space 0.55× as long as basal mandibular width; areolet ca. 1.80× as wide as height; fore wing with vein *1cu-a* vertical; hind wing with proximal abscissa of *CU* slightly inclivous and straight; metasoma mostly black, with posterior membranous section of first metasomal sternite, sternites II–VI and part of sternite VII white; ovipositor sheath ca. 1.25× as long as body, and ca. 3.60× as long as hind tibia.

#### Description.

**Figure 3.**
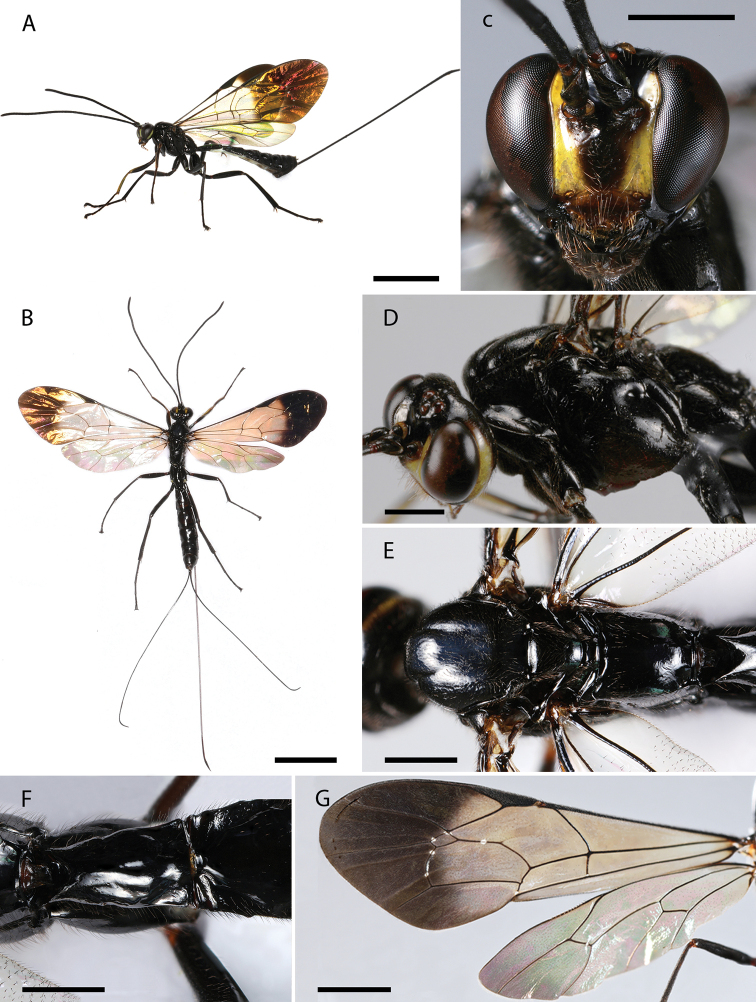
**A–G***Dolichomitus menai* sp. nov. (holotype female): **A** habitus in lateral view (*in vivo*) **B** habitus in dorsal view **C** head in frontal view **D** head and mesosoma in lateral view **E** mesosoma in dorsal view **F** first tergite in dorsal view **G** wings. Scale bars: 5.00 mm (**A, B**); 1.00 mm (**C, D, E, F**); 2.00 mm (**G**).

**Holotype female** (Fig. 3A–G). Approximate body length (without ovipositor): 12.50 mm; fore wing length: 12.30 mm.

***Head.*** Antenna with 31 flagellomeres, first flagellomere 4.50× as long as width. Gena smooth with setiferous punctures, in dorsal view somewhat rounded, 0.60× as long as eye, in frontal view almost straight and moderately constricted below eyes (Fig. 3C). Vertex smooth and shiny, with very isolated setiferous punctures. Posterior ocellus separated from eye 1.05× its maximum diameter. Distance between hind ocelli 0.65× maximum diameter of posterior ocellus. Face with fine, setiferous punctures. Clypeal sulcus slightly curved. Clypeus 2.70× as broad as medially long, almost flat. Clypeus with long parallels setae on its surface and small setae across all its margins. Anterior tentorial pits conspicuous. Malar space 0.55× as long as basal mandibular width. Mandible bidentate, 2.25× as long as basal width (Fig. 3C).

***Mesosoma.*** Pronotum polished, with fine and scattered setiferous punctures (Fig. 3D). Epomia present. Mesoscutum shiny, with moderately dense setiferous punctures. Notauli deep, reaching ca. 0.20 of length of mesoscutum. Mesopleuron shiny, with relatively dense setiferous punctures. Epicnemial carina strong. Metapleuron shiny, with scattered setiferous punctures, ca. 1.40× as long as height. Submetapleural carina strong, enlarged anteriorly, reaching ca. 0.55 of metapleuron length, its anterior end slightly curved up. Propodeum shiny, with fine and scattered setiferous punctures, denser anteriorly, in dorsal view 1.25× as long as medially wide. Propodeal spiracle elliptic, just above the pleural carina. Pleural carina strong, culminating posteriorly in a small propodeal crest. Hind leg with femur ca. 7.90× as long as height and ca. 0.70× as long as tibia. Fore wing with vein *1cu-a* interstitial to *M*&*Rs*; areolet ca. 1.80× as wide as height; vein *1cu-a* vertical, vein *2m-cu* slight curved. Hind wing with vein *M*+*CU* almost straight; vein *cu-a* ca. 2.50× as long as proximal abscissa of *CU*; vein *cu-a* reclivous and straight; proximal abscissa of *CU* slightly inclivous and straight; distal abscissa of *CU* present, reaching wing margin (Fig. 3G).

***Metasoma.*** Tergite I ca. 1.80× as long as posteriorly wide, shiny, with fine and relatively dense setiferous punctures, more extended laterally (Fig. 3F); spiracle near its anterior 0.40; dorsolateral carinae of first metasomal tergite weak, present on petiole and postpetiole. Posterior membranous section of first metasomal sternite ca. 0.25 of length of tergite. Tergite II ca. 1.20× as long as posteriorly wide, shiny, with fine and relatively dense setiferous punctures, more extended laterally and posteriorly; ovipositor slender, evenly down curved at distal 0.20, ca. 4.65× as long as hind tibia (Fig. 3A); apex of ovipositor with expanded area of lower valve bearing ca. 11 teeth, the most proximal 2 vertical; ovipositor sheath ca. 1.25× as long as body, and ca. 3.60× as long as hind tibia, bearing fine dense hairs which are ca. 1.35× as long as width of sheath.

***Color.*** Head mostly black with most of clypeus dark brown, anterior margin of clypeus, inner, frontal and outer orbit (but frons, stemmaticum and vertex, black) and temple yellow. Mesosoma entirely black shinning (Fig. 3B). Fore leg mostly black with ventral surfaces of femur and tibiae yellow. Mid and hind legs entirely black. Wings with iridescent rainbow colors with strongly contrasting apical darkened area that at least covers completely the fourth submarginal cell and third discal cell, pterostigma black (Fig. 3G). Metasoma mostly black shinning, with posterior membranous section of first metasomal sternite, sternites II–VI and part of sternite VII white (there are some randomly black spots on the sternites II–VII varying between the specimens) (Fig. 3A, B, F); ovipositor dark brown, darker on tip. Ovipositor sheath black.

**Male.** Unknown.

***Variation.*** There are some specimens with body length (12.50–19.00 mm) and wing length (12.30–18.50 mm).

#### Type material.

***Holotype.*** 1 ♀, Colombia, Jardín, Antioquia, El Alto, Mesenia-Paramillo nature reserve (1800m–3000m elevation), 5°29'45.8"N, 75°53'21.3"W, entomological net, 09–III–2019, coll. Mazariegos, L. (UNIANDES). ***Paratypes.*** 3 ♀♀, same locality, same collection method, 14–IV–2019, coll. Araujo, R. (UNIANDES); 2 ♀♀, same locality, same collection method, 15–IX–2019, coll. Rendon, U. (UNIANDES).

#### Distribution.

Colombia.

#### Etymology.

The specific epithet is in honor of Luis Fernando Mena for his continued support of the Mesenia-Paramillo nature reserve in the acquisition of forested areas for conservation. Mr. Mena is known for his support of important causes and has supported many NGO’s in Colombia that have an important social impact.

#### Biological note.

Host unknown.

#### Comments.

*Dolichomitus menai* sp. nov. is most similar to the *D. hypermeces* Townes, 1975, *D. irritator* (Fabricius, 1775) and *D. longicauda* Smith, 1877 mainly by black color of body. However, this new species differs mainly by having ovipositor sheath < 1.50× as long as body (except *D. irritator*) and the fore wing hyaline with strongly contrasting subapical darkened area, pterostigma black (entirely infumate and ovipositor sheath > 3.00× as long as body in *D. longicauda*; yellowish with pterostigma black in *D. irritator*; and brown with a broad pale yellowish brown band on apex and ovipositor sheath > 3.00× as long as body in *D. hypermeces*).

### Dolichomitus orejuelai

Taxon classificationAnimaliaHymenopteraIchneumonidae

Araujo & Pádua sp. nov.

438F8994-1E4D-5627-93F9-EA84DA70B06B

http://zoobank.org/7DCDBD61-6051-490B-B66E-F3DC1CFC13B3

Fig. 4A–G

#### Diagnosis.

*Dolichomitus orejuelai* sp. nov. may be distinguished from other Neotropical species by the combination of the following characteristics: head and mesosoma mostly reddish black; metasoma mostly yellowish brown with anterior half of tergite I dorsally, posterior margin of tergites II–V, a semicircular dorsal spot based on the anterior margin of tergite V, tergites VI–VIII reddish black; face with abundant setiferous punctures; malar space 0.30× as long as basal mandibular width; mandible bidentate, 1.40× as long as basal width; hind leg with femur ca. 5.50× as long as height; wings yellowish, pterostigma light brown; areolet not petiolated; dorsolateral carinae of first metasomal tergite present on petiole and stronger on postpetiole; posterior half of tergite II and tergites III–V densely and strongly punctuate; ovipositor sheath ca. 1.10× as long as body, and ca. 3.00× as long as hind tibia.

#### Description.

**Figure 4.**
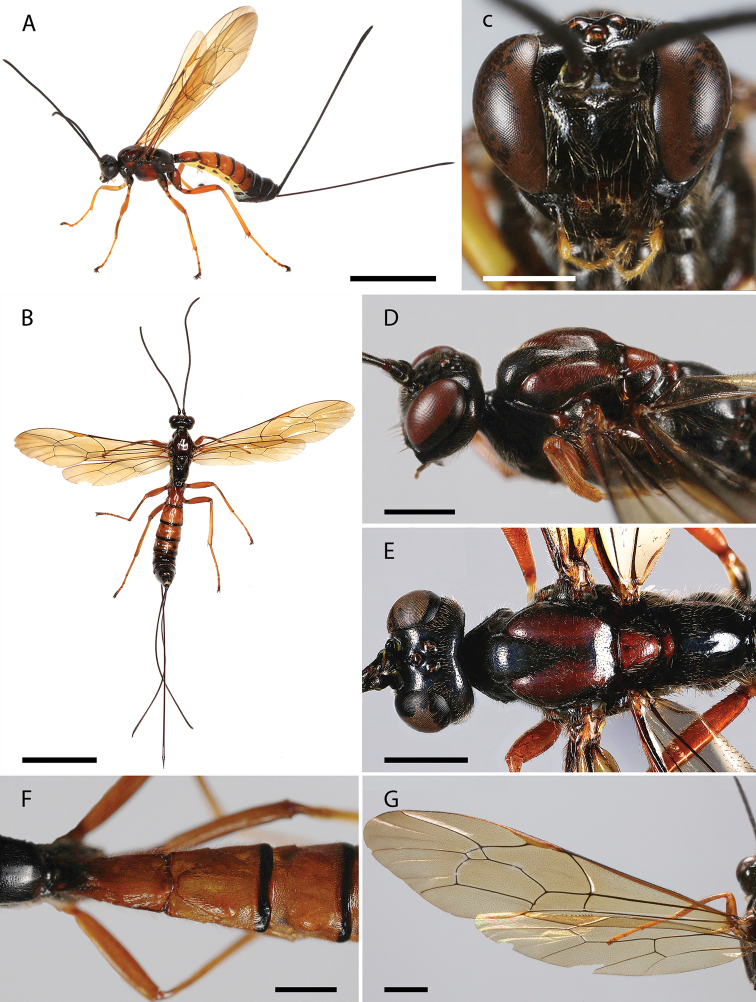
**A–G***Dolichomitus orejuelai* sp. nov. (holotype female): **A** habitus in lateral view (*in vivo*) **B** habitus in dorsal view **C** head in frontal view **D** head and mesosoma in dorsolateral view **E** mesosoma in dorsal view **F** tergites I–III in dorsal view **G** wings. Scale bars: 5.00 mm (**A, B**); 1.00 mm (**C, D, E, F**); 2.00 mm (**G**).

**Holotype female** (Fig. 4A–G). Approximate body length (without ovipositor): 12.30 mm; fore wing length: 11.15 mm.

***Head.*** Antenna with 32 flagellomeres, first flagellomere 4.50× as long as width. Gena smooth with setiferous punctures, in dorsal view somewhat rounded, 0.55× as long as eye (Fig. 4E), in frontal view almost straight and moderately constricted below eyes (Fig. 4C). Vertex smooth and shiny, with setiferous punctures. Posterior ocellus separated from eye 1.30× its maximum diameter. Distance between hind ocelli 0.85× maximum diameter of posterior ocellus. Occipital carina complete. Face with abundant setiferous punctures. Clypeal sulcus curved. Clypeus 3.20× as broad as medially long, almost flat. Clypeus with long parallels setae on its surface and small setae across all its margins. Anterior tentorial pits conspicuous. Malar space 0.30× as long as basal mandibular width. Mandible bidentate, 1.40× as long as basal width (front view).

***Mesosoma.*** Pronotum polished, with fine and scattered setiferous punctures. Epomia present. Mesoscutum shiny, with moderately dense setiferous punctures. Notauli deep, reaching ca. 0.40 of length of mesoscutum. Mesopleuron shiny, with relatively dense setiferous punctures. Epicnemial carina strong. Metapleuron shiny, with scattered setiferous punctures, ca. 1.65× as long as height. Submetapleural carina strong, enlarged anteriorly, reaching ca. 0.70 metapleuron length, its anterior end slightly curved up. Propodeum shiny, with fine and scattered setiferous punctures, denser laterally, in dorsal view 1.10× as long as medially wide. Propodeal spiracle elliptic, just above the pleural carina (Fig. 4D–E). Pleural carina complete and strong, culminating posteriorly in a small propodeal crest. Hind leg with femur ca. 5.50× as long as height and ca. 0.75× as long as tibia. Fore wing with vein *1cu-a* interstitial to *M*&*Rs*; areolet 1.45× as wide as height; vein *1cu-a* and vein *2m-cu* slight curved. Hind wing with vein *cu-a* ca. 2.25× as long as proximal abscissa of *CU*; vein *cu-a* reclivous and straight; proximal abscissa of *CU* vertical; distal abscissa of *CU* present, reaching wing margin (Fig. 4G).

***Metasoma.*** Tergite I ca. 1.90× as long as posteriorly wide, shiny, with fine and relatively dense setiferous punctures, more extended laterally (Fig. 4F); spiracle near its anterior 0.40; dorsolateral carinae of first metasomal tergite present on petiole and stronger on postpetiole. Posterior membranous section of first metasomal sternite ca. 0.40 of length of tergite. Tergite II ca. 1.10× as long as posteriorly wide, shiny, with fine and relatively dense setiferous punctures, more extended laterally; posterior half of tergite II and tergites III–V densely and strongly punctuate; ovipositor slender, evenly down curved at distal 0.20, ca. 4.35× as long as hind tibia (Fig. 4A); apex of ovipositor with expanded area of lower valve bearing ca. 7 teeth, the most proximal 2 vertical; ovipositor sheath ca. 1.10× as long as body, and ca. 3.00× as long as hind tibia, bearing fine dense hairs which are ca. 1.10× as long as width of sheath.

***Color.*** Head and antenna entirely reddish black. Mesosoma mostly reddish black with pronotal spiracle, two wide longitudinal stripes interrupted by notauli on mesoscutum, scutellum dorsally, metanotum dorsally, red. Fore, mid and hind legs mostly red, with coxa and trochanter (except posterior margin), reddish black. Wings yellowish, pterostigma light brown (Fig. 4G). Metasoma mostly yellowish brown with anterior half of tergite I dorsally, posterior margin of tergites II–V, a semicircular dorsal spot based on the anterior margin of tergite V, tergites VI–VIII, reddish black (Fig. 4A, B, F). Ovipositor dark brown and ovipositor sheath reddish black.

**Male.** Unknown.

***Variation.*** There are some specimens with body length (12.30–13.85 mm) and wing length (11.15–13.40 mm).

#### Type material.

***Holotype.*** 1 ♀, Colombia, Jardín, Antioquia, El Alto, Mesenia-Paramillo nature reserve (1800m–3000m elevation), 5°29'45.8"N, 75°53'21.3"W, entomological net, 24–IV–2019, coll. Mazariegos, L. (UNIANDES). ***Paratypes.*** 1 ♀, same locality, same collection method, 15–VIII–2019, coll. Jaramillo, J. (UNIANDES); 2 ♀♀, same locality, same collection method, 15–IX–2019, coll. Rendon, U. (UNIANDES).

#### Distribution.

Colombia.

#### Etymology.

The specific epithet is a tribute to Jorge Enrique Orejuela Gardner, National Geographic 2007 Buffet prize winner for his work over three decades in Colombia on conservation education, protected area management and sustainable development. His accomplishments include the establishment of the cloud forest nature reserve La Planada, also helped establish Utría and Gorgona Island national parks, and the Quindío Basin and Calima River nature reserves. His mentoring for the creation of the Mesenia-Paramillo nature reserve was key to the success of this conservation project.

#### Biological note.

Host unknown.

#### Comments.

*Dolichomitus orejuelai* sp. nov. is most similar to the Neotropical species *D. rufescens* (Cresson, 1865), *D. grilloi* Gauld, 1991, *D. flacissimus* Gauld, Ugalde & Hanson, 1998 and *D. bivittatus* Townes, 1975 mainly by color pattern reddish black or brown on the body. But this new species differs from *D. bivittatus* by having ovipositor sheath < 1.50× as long as body (> 3.50 in *D. bivittatus*) and differs from *D. flacissimus* by having fore wing yellowish with pterostigma light brown (infumate with pterostigma brown in *D. flacissimus*). Differs mainly from *D. grilloi* by having posterior ocellus separated from eye 1.30× its maximum diameter (0.80–0.90× in *D. grilloi*) and differs from *D. rufescens* by having metasoma mostly yellowish brown with anterior half of tergite I dorsally, posterior margin of tergites II–V, a semicircular dorsal spot based on the anterior margin of tergite V, tergites VI–VIII reddish black (entirely reddish in *D. rufescens*).

### Dolichomitus pimmi

Taxon classificationAnimaliaHymenopteraIchneumonidae

Araujo & Pádua sp. nov.

C798C036-54AF-5657-8402-347ADA4CDAC8

http://zoobank.org/374BC2BD-21FF-42D1-8766-6EFA8601FEC2

Fig. 5A–G

#### Diagnosis.

*Dolichomitus pimmi* sp. nov. may be distinguished from other Neotropical species by the combination of the following characteristics: general pattern of general color (orange yellow with various specifics black marks; wings yellowish with strongly contrasting apical darkened area, pterostigma light brown; areolet not petiolate; malar space 0.30× as long as basal mandibular width; mandible bidentate, 2.55× as long as basal width (front view); tergite I ca. 2.20× as long as posteriorly wide; ovipositor sheath ca. 0.90× as long as body, and ca. 3.00× as long as hind tibia.

#### Description.

**Figure 5.**
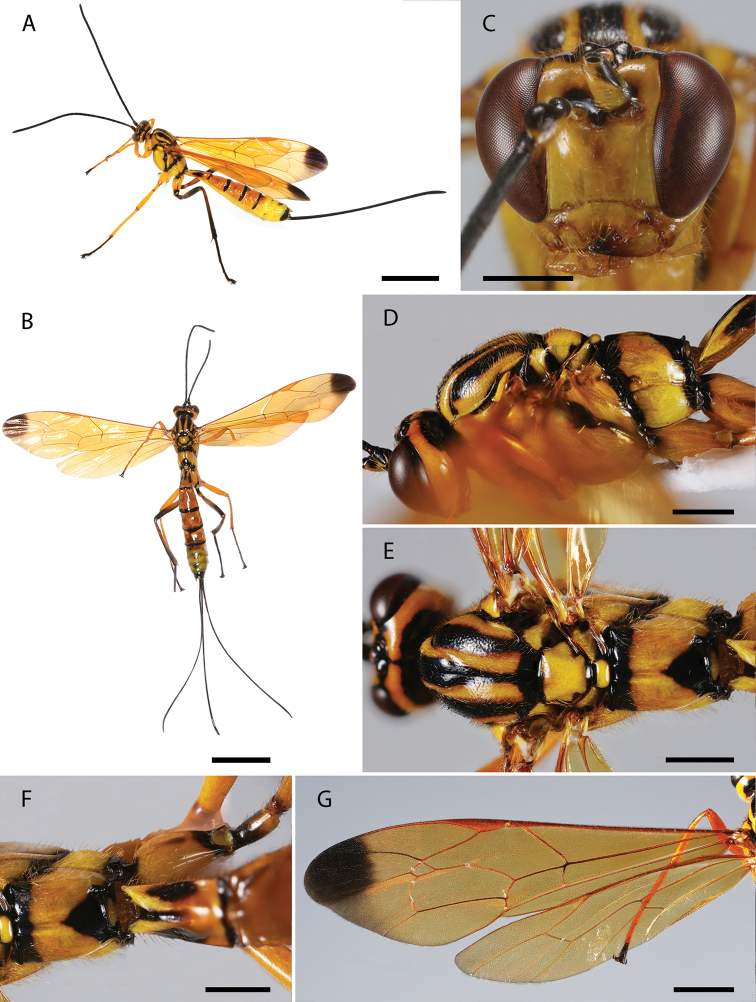
**A–G***Dolichomitus pimmi* sp. nov. (holotype female): **A** habitus in lateral view (*in vivo*) **B** habitus in dorsal view **C** head in frontal view **D** mesosoma in lateral view **E** mesosoma in dorsal view **F** first tergite in dorsal view **G** wings. Scale bars: 5.00 mm (**A, B**); 1.00 mm (**C, D, E, F**); 2.00 mm (**G**).

**Holotype female** (Fig. 5A–G). Approximate body length (without ovipositor): 15.55 mm; fore wing length: 14.70 mm.

***Head.*** Antenna with 32 flagellomeres, first flagellomere 4.50× as long as width. Gena smooth with setiferous punctures, in dorsal view somewhat rounded, 0.50× as long as eye, in frontal view almost straight and moderately constricted below eyes (Fig. 5C). Vertex smooth and shiny, with isolated setiferous punctures. Posterior ocellus separated from eye 1.40× its maximum diameter. Distance between hind ocelli 0.85× maximum diameter of posterior ocellus. Face with fine, setiferous punctures. Clypeal sulcus curved (Fig. 5C). Clypeus 3.45× as broad as medially long, almost flat. Clypeus with long parallels setae on its surface and small setae across all its margins. Anterior tentorial pits conspicuous. Malar space 0.30× as long as basal mandibular width. Mandible bidentate, 2.55× as long as basal width (front view).

***Mesosoma.*** Pronotum polished, with fine and scattered setiferous punctures. Epomia present. Mesoscutum shiny, with moderately dense setiferous punctures (Fig. 5D–E). Notauli deep, reaching ca. 0.30–0.40 of length of mesoscutum. Mesopleuron shiny, with relatively dense setiferous punctures. Epicnemial carina strong. Metapleuron shiny, with scattered setiferous punctures, ca. 1.70× as long as height. Submetapleural carina strong, enlarged anteriorly, reaching ca. 0.65 of metapleuron length, its anterior end slightly curved up. Propodeum shiny, with fine and scattered setiferous punctures, denser laterally, in dorsal view 1.20× as long as medially wide. Propodeal spiracle elliptic, just above the pleural carina. Pleural carina complete and strong, culminating posteriorly in a small propodeal crest. Hind leg with femur ca. 6.60× as long as height and ca. 0.70× as long as tibia. Fore wing with vein *1cu-a* more or less interstitial to *M*&*Rs*; areolet 1.50× as wide as height; vein *1cu-a* and vein *2m-cu* slightly curved. Hind wing with vein *cu-a* ca. 2.60× as long as proximal abscissa of *CU*; vein *cu-a* reclivous and straight; proximal abscissa of *CU* vertical; distal abscissa of *CU* present, reaching wing margin (Fig. 5G).

***Metasoma.*** Tergite I ca. 2.20× as long as posteriorly wide, shiny, with fine and relatively dense setiferous punctures, more extended laterally (Fig. 5F); spiracle near its anterior 0.35; dorsolateral carinae of first metasomal tergite weak, present on petiole and postpetiole. Posterior membranous section of first metasomal sternite ca. 0.30 of length of tergite. Tergite II ca. 1.20× as long as posteriorly wide, shiny, with fine and relatively dense setiferous punctures, more extended laterally and posteriorly; ovipositor slender, evenly down curved at distal 0.30, ca. 3.40× as long as hind tibia (Fig. 5A); upper valve of ovipositor smooth; apex of ovipositor with expanded area of lower valve bearing ca. 10 teeth, the most proximal 3 subvertical; ovipositor sheath ca. 0.90× as long as body, and ca. 3.00× as long as hind tibia, bearing fine dense hairs which are ca. 1.10× as long as width of sheath.

***Color.*** Head mostly orange yellow with mandibles apically, frons, stemmaticum (extending to outer orbit dorsally), a longitudinal stripe on vertex, dorsal half of occipital carinae, scape dorsally, pedicel and flagellum black. Mesosoma mostly orange yellow with pronotal collar dorsally (dorsally extending to pronotum), posterior margin of pronotum, tegula dorsally, subtegular ridge, lateral and central longitudinal stripes on mesoscutum, scuto-scutellar groove, ventral and posterior margin of scutellum, posterior margin of axilla, metanotum posteriorly, epicnemium ventrally, anterior and dorsal margin of mesopleuron, anterior margin of mesepisternum, anterior and posterior margin of metapleuron, a narrow strip over the submetapleural carina, anterior and posterior margin of propodeum, a triangle mark dorsally based on the posterior margin that projects to the center of propodeum, black. Fore and mid legs mostly orange yellow with a dorsal mark on the anterior margin of coxa, tarsi V on fore and mid leg and tarsal claws, black. Mid leg with tarsi II–IV dark brown. Hind leg mostly dark brown (infuscate) with the coxa orange yellow; posterior half of trochanter and anterior half of femur dark yellow; anterior and posterior margin of coxa, posterior margin of tibia and tarsus, black (Fig. 5A, B, F). Wings yellowish with strongly contrasting apical darkened area that covers only the distal half of fourth submarginal cell, pterostigma light brown (Fig. 5G). Metasoma mostly orange yellow with anterior half of tergite I, posterior half of tergite V, tergites VI–VII and posterior margin of tergite VIII yellow. Lateral spots and an anterior dorsal longitudinal stripe on tergite I, posterior margin of tergites I–IV, tergite VIII black. Anterior margin of tergites VI and VII with a dark brown spot dorsally. Ovipositor dark brown and ovipositor sheath black.

**Male.** Unknown.

***Variation.*** There are some specimens with body length (11.80–15.55 mm) and wing length (11.15–14.70 mm).

#### Type material.

***Holotype.*** 1 ♀, Colombia, Jardín, Antioquia, El Alto, Mesenia-Paramillo nature reserve (1800m–3000m elevation), 5°29'45.8"N, 75°53'21.3"W, entomological net, 09–III–2019, coll. Mazariegos, L. (UNIANDES). ***Paratypes.*** 1 ♀, same locality, same collection method, 14–IV–2019, same collector (UNIANDES); 1 ♀, same locality, same collection method, 15–IX–2019, coll. Rendon, U. (UNIANDES).

#### Distribution.

Colombia.

#### Etymology.

The specific epithet is in honor of Stuart Pimm, Doris Duke Chair of Conservation Ecology in the Nicholas School of the Environment at Duke University. Winner of the 2006 Heineken Prize for Environmental Sciences, awardee of the Tyler Prize for Environmental Achievement in 2010, and recipient of the 2019 International Cosmos Prize – among the most prestigious honors in the environmental field – for his research on endangered species and his work to help reverse species’ declines by protecting their shrinking habitats. His support of the Mesenia-Paramillo nature reserve conservation project to restore areas and reconnect forest fragments has been invaluable.

#### Biological note.

Host unknown.

#### Comments.

*Dolichomitus pimmi* sp. nov. is most similar to *D. mariajosae* sp. nov. mainly by the pattern color of the body yellowish with black marks and the fore wing with a strongly contrasting apical darkened area, but this new species differs mainly by the fore wing yellowish with pterostigma light brown, hind wing with proximal abscissa of *CU* vertical, ovipositor ca. 3.40× as long as hind tibia and ovipositor sheath ca. 3.00× as long as hind tibia (fore wing hyaline with pterostigma dark brown, hind wing with proximal abscissa of *CU* inclivous, ovipositor ca. 4.40× as long as hind tibia and ovipositor sheath ca. 4.20× as long as hind tibia in *D. mariajosae* sp. nov.).

### Dolichomitus rendoni

Taxon classificationAnimaliaHymenopteraIchneumonidae

Araujo & Pádua sp. nov.

E1974829-D41F-5146-96D8-4474404977EB

http://zoobank.org/A42CEA69-EC56-4F69-954E-56B92EAB9839

Fig. 6A–G

#### Diagnosis.

*Dolichomitus rendoni* sp. nov. may be distinguished from other Neotropical species by the combination of the following characteristics: malar space 0.35× as long as basal mandibular width; mesosoma mostly red with the tegula white; wings yellowish, pterostigma dark brown; areolet slightly petiolate; fore leg with a white concavity on it postero-dorsal margin; fore and mid legs mainly white; hind wing with vein *cu-a* ca. 1.20× as long as proximal abscissa of *CU*; metasoma mostly reddish black with ventro-lateral spots on tergites III–IV, lateral of tergites V–VIII red (except for the posterior margin of tergites V and VI laterally reddish black); posterior margin of tergite I–VII with a white band dorsally (small and narrow on tergite I); posterior membranous section of first metasomal sternite ca. 0.60 of length of tergite; ovipositor sheath ca. 0.90× as long as body, and ca. 2.90× as long as hind tibia.

#### Description.

**Figure 6.**
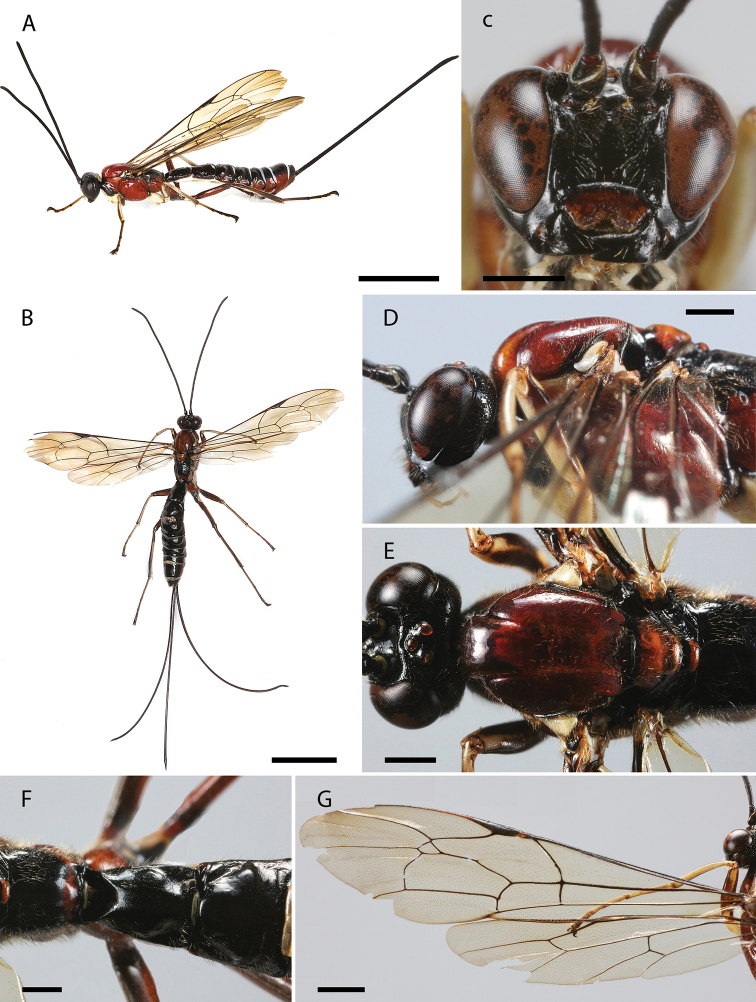
**A–G***Dolichomitus rendoni* sp. nov. (holotype female): **A** habitus in lateral view (*in vivo*) **B** habitus in dorsal view **C** head in frontal view **D** head and mesosoma in lateral view **E** head and mesosoma in dorsal view **F** first tergite in dorsal view **G** wings. Scale bars: 5.00 mm (**A, B**); 1.00 mm (**C, D, E, F**); 2.00 mm (**G**).

**Holotype female** (Fig. 6A–G). Approximate body length (without ovipositor): 13.50 mm; fore wing length: 12.00 mm.

***Head.*** Antenna with 31–34 flagellomeres, first flagellomere 4.20× as long as width. Gena smooth with setiferous punctures, in dorsal view somewhat rounded, 0.55× as long as eye (Fig. 6E), in frontal view almost straight below eyes (Fig. 6C). Vertex smooth and shiny, with setiferous punctures. Posterior ocellus separated from eye 1.10× its maximum diameter. Distance between hind ocelli 0.95× maximum diameter of posterior ocellus. Occipital carina complete. Face with abundant setiferous punctures. Clypeal sulcus curved. Clypeus 3.15× as broad as medially long, almost flat. Clypeus with sparse parallels setae on its surface. Anterior tentorial pits conspicuous. Malar space 0.35× as long as basal mandibular width. Mandible bidentate, 1.40× as long as basal width (front view).

***Mesosoma.*** Pronotum polished, with fine and scattered setiferous punctures. Epomia present. Mesoscutum shiny, with sparse setiferous punctures. Notauli deep, reaching ca. 0.40 of length of mesoscutum. Mesopleuron shiny, with relatively dense setiferous punctures. Epicnemial carina strong. Metapleuron shiny, with relatively dense setiferous punctures, ca. 1.30× as long as height. Submetapleural carina strong, enlarged anteriorly, reaching ca. 0.65 metapleuron length, its anterior end slightly curved up. Propodeum shiny, with fine and scattered setiferous punctures, denser laterally, in dorsal view 1.05× as long as medially wide. Propodeal spiracle elliptic (Fig. 6D–E). Pleural carina complete and strong. Hind leg with femur ca. 5.60× as long as height and ca. 0.80× as long as tibia. Fore wing with vein *1cu-a* interstitial to *M*&*Rs*; areolet slightly petiolate, 1.60× as long as height; vein *1cu-a* and vein *2m-cu* slightly curved. Hind wing with vein *cu-a* ca. 1.20× as long as proximal abscissa of *CU*; vein *cu-a* reclivous and straight; proximal abscissa of *CU* vertical; distal abscissa of *CU* present, reaching wing margin.

***Metasoma.*** Tergite I ca. 1.40× as long as posteriorly wide, shiny, with fine and relatively dense setiferous punctures, more extended laterally (Fig. 6F); spiracle near its anterior 0.45; dorsolateral carinae of first metasomal tergite present on petiole and stronger on postpetiole. Posterior membranous section of first metasomal sternite ca. 0.60 of length of tergite. Tergite II ca. 1.20× as long as posteriorly wide, shiny, with fine and relatively dense setiferous punctures, more extended laterally. Ovipositor slender, evenly down curved at distal 0.12, ca. 3.60× as long as hind tibia (Fig. 6A); apex of ovipositor with expanded area of lower valve bearing ca. 10 teeth, the most proximal are 2 vertical, followed by 2 subvertical; ovipositor sheath ca. 0.90× as long as body, and ca. 2.90× as long as hind tibia, bearing fine dense hairs which are ca. 0.90× as long as width of sheath.

***Color.*** Head and antenna entirely reddish black. Mesosoma mostly red with propleuron, pronotal collar, anterior margin of pronotum, two marks on the lateral margins of mesoscutum (just above tegula), subtegular ridge, scuto-scutellar groove, axilla, metanotum anteriorly, a narrow mark on the ventro-anterior margin of epicnemium, a narrow mark on the ventral half of mesopleural furrow, a narrow mark on the ventral margin of mesepisternum, propodeum reddish black. Tegula white, with posterior margin infuscate. Fore leg mostly white with the dorsal surface of femur (except for a white concavity on it postero-dorsal margin), ventral surface of tibia and tarsus reddish brown. Mid leg mostly white with the dorsal surface of femur (except for a white spot on it postero-dorsal margin), ventral surface of tibia, tarsi II–V reddish brown (tarsus I infuscate). Hind leg mostly reddish brown with the ventral surface of trochanter and trochantellus, posterior 0.70 of the dorsal surface of tibia white; coxa and ventral surface of femur red (Fig. 6A, B, F). Wings yellowish, pterostigma dark brown (Fig. 6G). Metasoma mostly reddish black with ventro-lateral spots on tergites III–IV, lateral of tergites V–VIII red (except for the posterior margin of tergites V and VI laterally reddish black). Posterior margin of tergite I–VII with a white band dorsally (small and narrow on tergite I). Ovipositor dark brown and ovipositor sheath reddish black.

**Male.** Unknown.

***Variation.*** There are some paratypes with body length (17.50 mm), fore wing length (15.95 mm) and the coloration (posterior margin of propodeum red; width of the dorsal white bands narrower on the posterior margin of tergites II–IV).

#### Type material.

***Holotype.*** 1 ♀, Colombia, Jardín, Antioquia, La Lucrecia, Mesenia-Paramillo nature reserve (2400m elevation), 5°30'50.61"N, 75°50'32.02"W, entomological net, 06–I–2020, coll. Jaramillo, J. (UNIANDES). ***Paratype.*** 1 ♀, same locality, same collection method, 15–IX–2019, coll. Rendon, U. (UNIANDES).

#### Distribution.

Colombia.

#### Etymology.

The specific epithet is in honor of Ubiel Rendon, park ranger at the Mesenia-Paramillo nature reserve. A La Mesenia village native and once an avid hunter, his knowledge of the surrounding forests has been key for monitoring wildlife and helping with long-term studies using camera traps. He has made several important contributions to the scientific world, finding multiple new species of amphibians, reptiles and orchids at the reserve, including this Darwin wasp named in his honor.

#### Biological note.

Host unknown.

#### Comments.

*Dolichomitus rendoni* sp. nov. is most similar to *D. orejuelai* sp. nov. mainly by the pattern color reddish black on head, antenna, propleuron, pronotal collar, anterior margin of pronotum and propodeum dorsally, besides the yellowish wings. But this new species differs by having the areolet slightly petiolate, hind wing with vein *cu-a* ca. 1.20× as long as proximal abscissa of *CU*, pterostigma dark brown and the pattern color mostly white on fore and mid legs (areolet not petiolate, hind wing with vein *cu-a* ca. 2.25× as long as proximal abscissa of *CU*, pterostigma light brown and the pattern color mostly red on fore and mid legs in *D. orejuelai* sp. nov.).

## Supplementary Material

XML Treatment for Dolichomitus

XML Treatment for Dolichomitus mariajosae

XML Treatment for Dolichomitus menai

XML Treatment for Dolichomitus orejuelai

XML Treatment for Dolichomitus pimmi

XML Treatment for Dolichomitus rendoni
